# *Cx3cr1* deficiency in mice attenuates hepatic granuloma formation during acute schistosomiasis by enhancing the M2-type polarization of macrophages

**DOI:** 10.1242/dmm.018242

**Published:** 2015-07-01

**Authors:** Lin Ran, Qilin Yu, Shu Zhang, Fei Xiong, Jia Cheng, Ping Yang, Jun-Fa Xu, Hao Nie, Qin Zhong, Xueli Yang, Fei Yang, Quan Gong, Michal Kuczma, Piotr Kraj, Weikuan Gu, Bo-Xu Ren, Cong-Yi Wang

**Affiliations:** 1 Department of Molecular Biology, Medical College of Yangtze University, 1 Nanhuan Road, Jingzhou, Hubei 434023, China; 2 The Center for Biomedical Research, Tongji Hospital, Tongji Medical College, Huazhong University of Science and Technology, 1095 Jiefang Avenue, Wuhan 430030, China; 3 Department of Clinical Immunology, Institute of Laboratory Medicine, Guangdong Medical College, No. 1 Xincheng Road, Dongguan 523808, China; 4 Clinical and Molecular Immunology Research Center, Medical College of Yangtze University, 1 Nanhuan Road, Jingzhou, Hubei 434023, China,; 5 Department of Immunology, Medical College of Yangtze University, 1 Nanhuan Road, Jingzhou, Hubei 434023, China; 6 The Center for Biotechnology and Genomic Medicine, Georgia Regents University, 1120 15th Street, Augusta, GA 30912, USA; 7 Department of Orthopedic Surgery and BME, Campbell-Clinic, University of Tennessee, Health Science Center, Memphis, TN 38163, USA

**Keywords:** CX3CR1, Schistosomiasis, Granuloma formation, Macrophage, STAT-6, PPAR-γ

## Abstract

Acute schistosomiasis is characterized by pro-inflammatory responses against tissue- or organ-trapped parasite eggs along with granuloma formation. Here, we describe studies in *Cx3cr1^−/−^* mice and demonstrate the role of Cx3cr1 in the pathoetiology of granuloma formation during acute schistosomiasis. Mice deficient in *Cx3cr1* were protected from granuloma formation and hepatic injury induced by *Schistosoma japonicum* eggs, as manifested by reduced body weight loss and attenuated hepatomegaly along with preserved liver function. Notably, *S. japonicum* infection induced high levels of hepatic *Cx3cr1* expression, which was predominantly expressed by infiltrating macrophages. Loss of *Cx3cr1* rendered macrophages preferentially towards M2 polarization, which then led to a characteristic switch of the host immune defense from a conventional Th1 to a typical Th2 response during acute schistosomiasis. This immune switch caused by *Cx3cr1* deficiency was probably associated with enhanced STAT6/PPAR-γ signaling and increased expression of indoleamine 2,3-dioxygenase (IDO), an enzyme that promotes M2 polarization of macrophages. Taken together, our data provide evidence suggesting that CX3CR1 could be a viable therapeutic target for treatment of acute schistosomiasis.

## Abstract (precis)

**Highlighted Article:** A reduction in CX3CR1 signaling provides protection for mice against pro-inflammatory responses and hepatic granuloma formation during acute schistosomiasis.

## INTRODUCTION

A characteristic pathological manifestation of schistosomiasis is the granulomatous response against tissue- or organ-trapped parasite eggs (ova) (Pearce and MacDonald, 2002). In particular, the formation of hepatic egg granulomas and secondary hepatic fibrosis are the primary cause of death in schistosomiasis (Vennervald and Dunne, 2004). Soluble egg antigen (SEA) originating from the eggs of *Schistosoma japonicum* is potent enough to evoke pro-inflammatory responses by recruiting macrophages into the liver, which then initiate granuloma formation to limit the immune responses against SEA to the location of the trapped egg in the liver (Burke et al., 2010; Qiu et al., 2001; Shimaoka et al., 2007). Given that macrophages serve as a bridge to link innate immunity to adaptive immune responses, they have now been recognized to play a crucial role in the pathogenesis of granuloma formation during the course of schistosomiasis (Behrens, 2008; Christophi et al., 2009; Gordon and Martinez, 2010; Noel et al., 2004; Ragheb and Boros, 1989).

In general, praziquantel is thus far the best therapeutic choice for treatment of schistosomiasis, although recent studies have consistently raised concerns about the development of parasite praziquantel resistance. Furthermore, schistosomes possess the capability to evade the immune system of the host, which allows them to survive intravascularly for many years in the face of an ongoing antiparasite immune response by the infected host (Pearce and MacDonald, 2002). As a result, sustained aggravation of hepatic granulomatous inflammatory responses and subsequent fibrosis are commonly noted in certain patients, even when efficacious antiparasitic drugs are administered (Cioli and Pica-Mattoccia, 2003). Therefore, a better understanding of the pathoetiologies underlying granuloma formation during the course of schistosome infection is essential to develop novel effective therapeutic strategies for prevention and treatment of hepatic fibrosis.

Previous studies have suggested that chemokines and their receptors not only coordinate inflammatory infiltration but also modulate the function of resident immune cells in the setting of tissue and/or organ injury or infection. In particular, CX3CR1 has been implicated in the pathogenesis of rheumatoid arthritis, glomerulonephritis, atopic dermatitis, psoriasis, Crohn's disease and atherosclerosis (Ishida et al., 2008). More recently, several studies have provided evidence supporting the idea that the expression of Cx3cr1 on monocytes or macrophages promotes wound healing and fibrotic processes (Martins-Green et al., 2013). Based on these observations, we thus hypothesized that CX3CR1 signaling in infiltrating macrophages could play a crucial role in the formation of hepatic egg granulomas after schistosome infection. To test this hypothesis, B6 mice deficient in *Cx3cr1* were infected with cercariae of *S. japonicum*. Our data demonstrate that loss of *Cx3cr1* signaling significantly protected mice from hepatic granuloma formation along with preserved liver function.

**TRANSLATIONAL IMPACT**

**Clinical issue**

Schistosomiasis is a parasitic disease that affects more than 210 million people worldwide. Its major pathology is the induction of a pro-inflammatory response against parasite eggs trapped in tissues or organs, which leads to the formation of granulomas (nodules of immune system cells that wall off and contain foreign bodies). In general, praziquantel is the best therapeutic choice for treatment of infections with all major schistosome species. However, praziquantel is only effective against adult worms and requires the presence of a mature antibody response to the parasite. Furthermore, schistosomes possess the capability to evade the immune system of the host. As a result, sustained aggravation of hepatic granulomatous inflammatory responses and subsequent fibrosis are commonly noted in some individuals affected by schistosomiasis even when efficacious antiparasitic drugs are administered.

**Results**

Recently, it has been reported that the expression of the chemokine Cx3cr1 on monocytes and macrophages promotes wound healing and fibrotic processes. In this study, therefore, the authors test the hypothesis that CX3CR1 signaling in infiltrating macrophages plays a crucial role in the formation of hepatic granulomas after schistosome infection using mice infected with *Schistosoma japonicum*, a well-established model of human schistosomiasis. The authors report that mice deficient in *Cx3xr1* are protected from granuloma formation and hepatic injury induced by *S. japonicum* eggs, as manifested by a reduced loss of body weight, attenuated hepatomegaly and preservation of liver function. Notably, *S. japonicum* infection induced high levels of Cx3cr1 expression in the liver, predominantly by infiltrating macrophages. Moreover, loss of *Cx3cr1* directed macrophages preferentially towards M2 polarization. This, in turn, led to a characteristic switch of host immune defense in the setting of acute schistosomiasis from a conventional Th1 to a typical Th2 response. Finally, the authors show that this immune switch was associated with enhanced STAT6/PPAR-γ signaling and increased expression of IDO, a tryptophan-metabolizing enzyme that promotes M2 polarization of macrophages.

**Implications and future directions**

These findings support the hypothesis that Cx3cr1 signaling in infiltrating macrophages is linked to the formation of hepatic egg granulomas after schistosome infection in mice. These findings therefore suggest that it might be possible to attenuate hepatic granuloma formation in people infected with schistosomes by suppressing the expression of CX3CR1. Thus, CX3CR1 could be a viable therapeutic target for the treatment of individuals with acute schistosomiasis.

## RESULTS

### Loss of *Cx3cr1* protects mice against hepatic granuloma formation

**Fig. 1.**
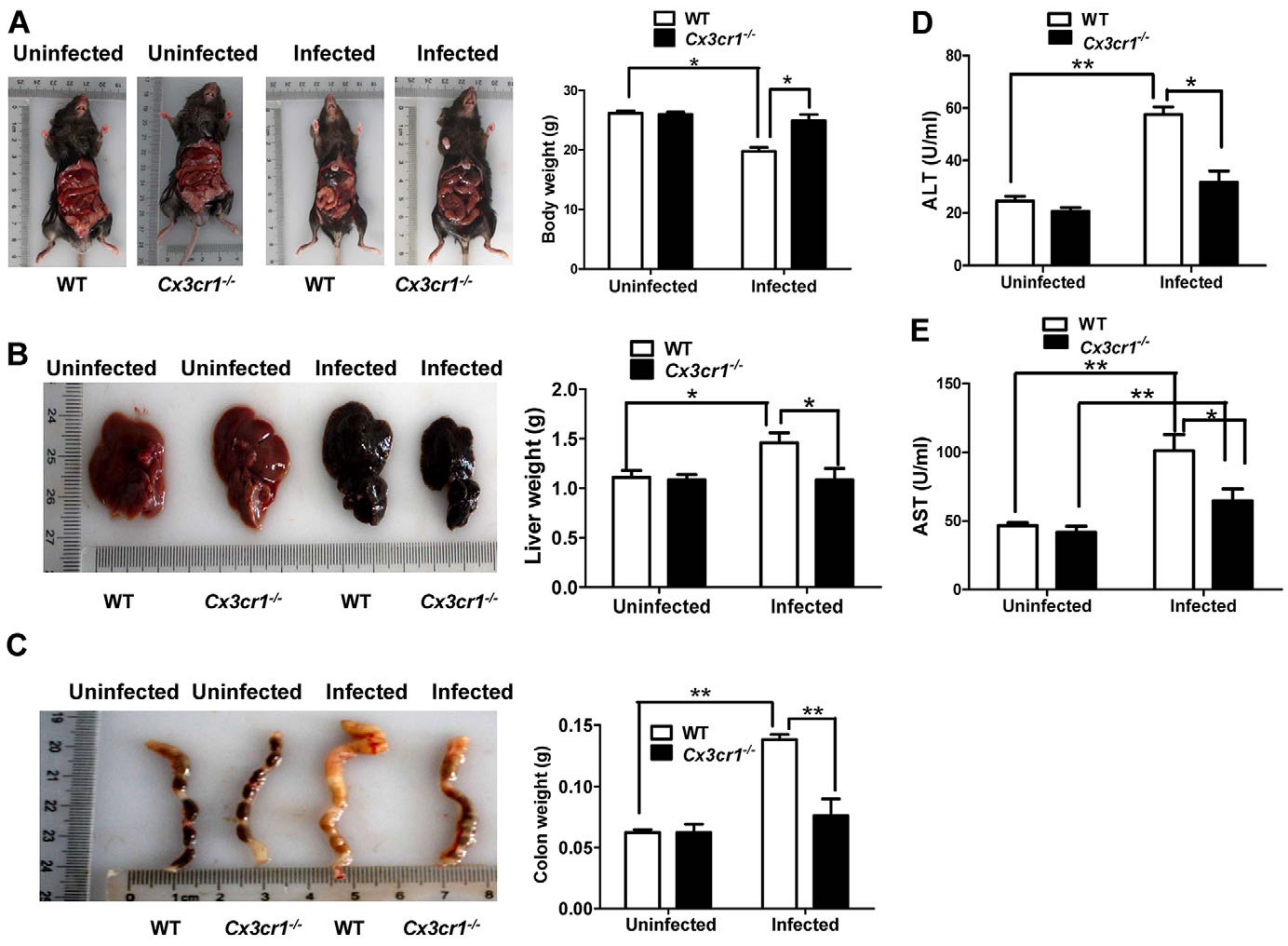
**Pathological manifestations 8 weeks after *S. japonica* infection.** (A) Body weight changes after *S. japonica* infection. (B) Liver weight changes. (C) Colonic swelling and damage after *S. japonica* infection. (D) ALT levels after *S. japonica* infection. (E) Results for AST levels. *Cx3cr1^−/−^* mice were significantly protected from hepatic injury and functional impairment mediated by *S. japonicum* infection as manifested by the reduced body weight loss, attenuated hepatomegaly along with reserved liver function. A total of 15 mice were analyzed in each study group. *P<0.05; **P<0.01.

Wild-type (WT) mice and *Cx3cr1^−/−^* mice were percutaneously infected with 30 cercariae of *S. japonicum*, and the mice were sacrificed 8 weeks after infection. We first noted that *S. japonicum* infection resulted in a significant reduction in body weight in WT mice, and in sharp contrast, *Cx3cr1^−/−^* mice generally looked healthy as manifested by no statistical change in terms of body weight (Fig. 1A). Furthermore, WT mice displayed a marked increase in liver weight as manifested by hepatomegaly (Fig. 1B), and a similar increase in the colonic weight was also noted in WT mice after *S. japonicum* infection as compared with that of *Cx3cr1^−/−^* mice (Fig. 1C).

**Fig. 2.**
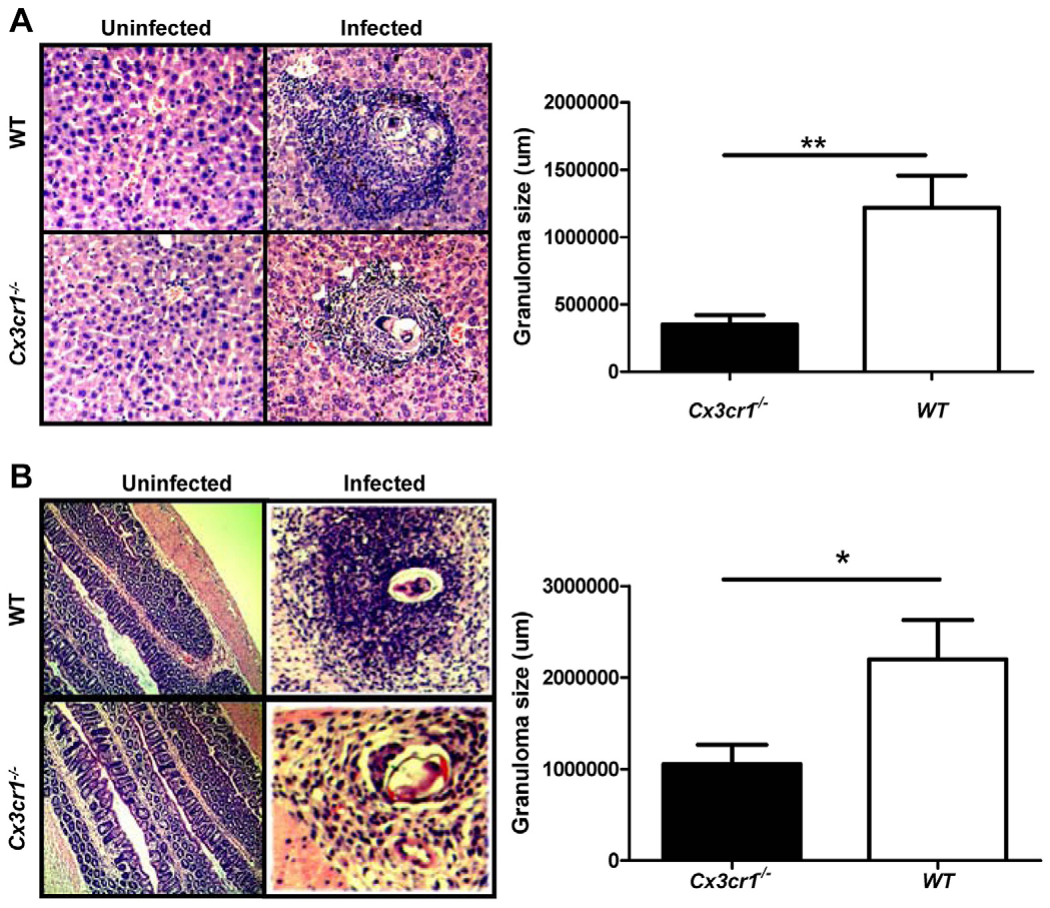
***S. japonica* egg-induced granuloma formation.** (A) Analysis of granuloma size in the liver. (B) Analysis of colonic granuloma size. The number of granulomas and their size in the sections were analyzed in both WT and *Cx3cr1^−/−^* mice before and 8 weeks after *S. japonica* infection. The graphs show results after *S. japonica* infection. Eight mice were analyzed in each study group. *P<0.05; **P<0.01.

Next, we sought to examine the impact of *S. japonicum* infection on liver function by assaying the activities of alanine transaminase (ALT) and aspartate transaminase (AST). Both WT and *Cx3cr1^−/−^* mice manifested an increase in ALT and AST activity 8 weeks after cercariae infection. WT mice showed significantly higher levels of ALT (Fig. 1D) and AST (Fig. 1E) as compared with that in *Cx3cr1^−/−^* mice, demonstrating that *Cx3cr1* deficiency protected mice from the hepatic injury caused by *S. japonicum* infection. H&E staining of liver sections was then carried out to assess granuloma formation. In accordance with the above results, a significantly smaller average hepatic granuloma size was observed in *Cx3cr1^−/−^* mice as compared with that of WT control mice (122,118±2376 μm^2^ versus 35,155±697 μm^2^, mean±s.e.m., *P*<0.001, Fig. 2A), and similar results were also noted in the colon (Fig. 2B). Collectively, our data support the idea that loss of *Cx3cr1* provides protection for mice against hepatic granuloma formation and functional impairment induced by *S. japonicum* eggs.

**Fig. 3.**
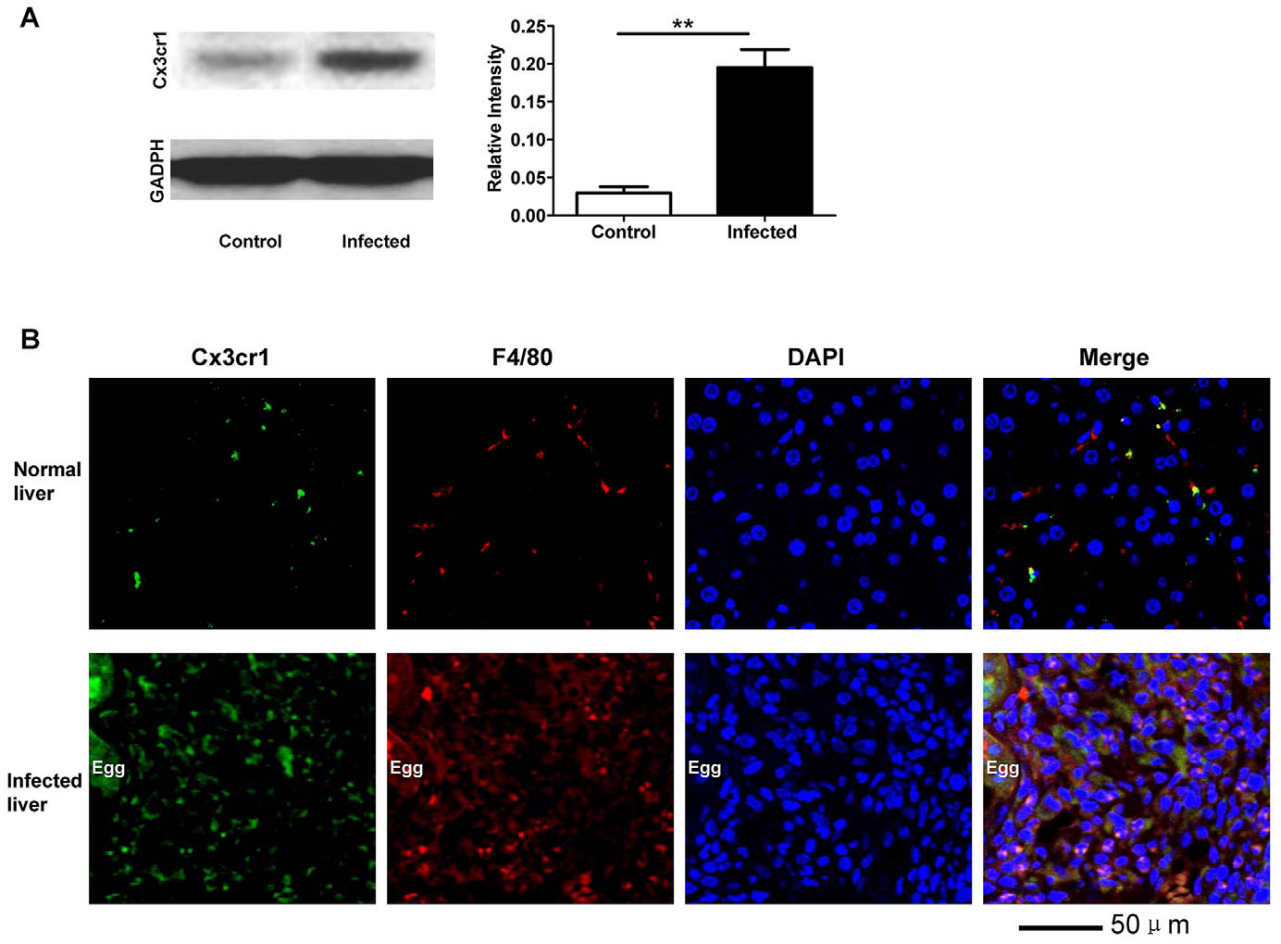
***S. japonica* infection induces high levels of hepatic Cx3cr1 expression.** (A) Western blot analysis of Cx3cr1 expression in the liver lysates 8 weeks after *S. japonica* infection. **P<0.01. (B) Co-immunostaining of Cx3cr1 and F4/80 in the liver sections 8 weeks after *S. japonica* infection. Substantial macrophage infiltration along with high levels of Cx3cr1 expression was noted after *S. japonica* infection as manifested by the colocalization of Cx3cr1 and F4/80.

### *S. japonicum* infection induces high levels of hepatic Cx3cr1 expression

To further demonstrate the role of Cx3cr1 in hepatic granuloma formation induced by *S. japonicum* eggs, we then examined hepatic Cx3cr1 expression in WT mice 8 weeks after cercariae infection; age matched WT mice in the absence of *S. japonicum* infection served as controls. Interestingly, only low levels of Cx3cr1 expression were detected in the liver under physiological conditions. However, *S. japonicum* infection induced almost a 7-fold increase in hepatic Cx3cr1 expression (Fig. 3A). To confirm these data, we conducted immunostaining of liver sections for Cx3cr1 along with F4/80, a specific marker for macrophages. As shown in Fig. 3B, Cx3cr1 was almost undetectable in the sections originating from normal mice, and only some sporadic F4/80-positive macrophages were observed. In sharp contrast, a high intensity of immunofluorescence specific for Cx3cr1 was noted within the granuloma in the sections derived from *S. japonicum*-infected mice, and a significant accumulation of F4/80-positive macrophages within the granulomatous area was noted. In particular, Cx3cr1 immunofluorescence colocalized with F4/80 staining, suggesting that Cx3cr1 was expressed predominantly by the infiltrating macrophages. Of note, although macrophages were the major type of infiltrating immune cells, other inflammatory cells such as neutrophils, lymphocytes and eosinophils were also detected in the granulomas (data not shown).

**Fig. 4.**
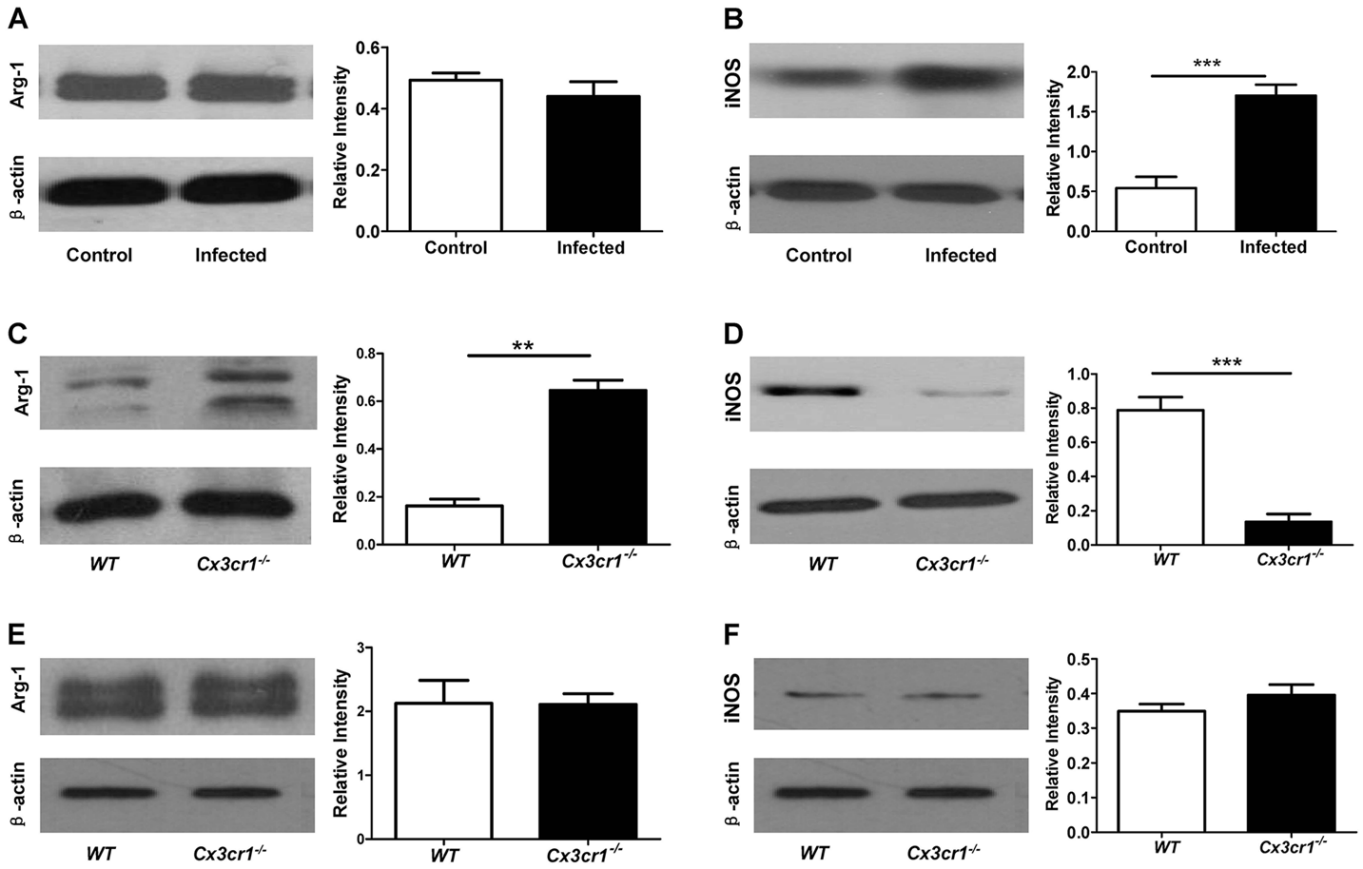
**Western blot analysis of hepatic Arg-1 and iNOS expression during acute schistosomiasis.** (A) *S. japonica* infection (8 weeks) did not result in a perceptible change in the expression of Arg-1 in the liver. (B) *S. japonica* infection (8 weeks) induced high levels of iNOS expression in the liver. (C) Loss of *Cx3xr1* significantly induced Arg-1 expression in the setting of *S. japonica* infection (8 weeks). (D) *Cx3cr1* deficiency significantly attenuated *S. japonica*-induced iNOS expression in the liver. Four mice were analyzed for each study group. **P<0.01, ***P<0.001. (E) There was no significant difference in the expression of Arg-1 in the liver between WT and *Cx3cr1^−/−^* mice before *S. japonicum* infection. (F) No significant difference in terms of hepatic iNOS expression between WT and *Cx3cr1^−/−^* mice was observed before *S. japonicum* infection.

### *Cx3cr1^−/−^* mice manifest a Th2 response against liver-trapped *S. japonicum* eggs

To dissect the mechanisms by which *Cx3cr1* deficiency attenuates hepatic granuloma formation after *S. japonicum* infection, we first examined arginase-1 (Arg-1) and inducible NO synthase (iNOS) expression in the liver, because enhanced Arg-1 expression is associated with a Th2 immunity to liver-trapped *S. japonicum* eggs, whereas a Th1-mediated response is a feature of increased iNOS expression (Zhang et al., 2001). Interestingly, no significant difference in terms of hepatic Arg-1 expression between *S. japonicum*-infected mice and control mice was observed (Fig. 4A). However, more than a 2-fold higher iNOS expression was noted in *S. japonicum*-infected WT mice as compared with that of uninfected WT control mice (Fig. 4B), confirming that hepatic granuloma formation in acute schistosomiasis is predominantly mediated by a Th1-based immune response rather than a Th2-related immunity in WT mice.

**Fig. 5.**
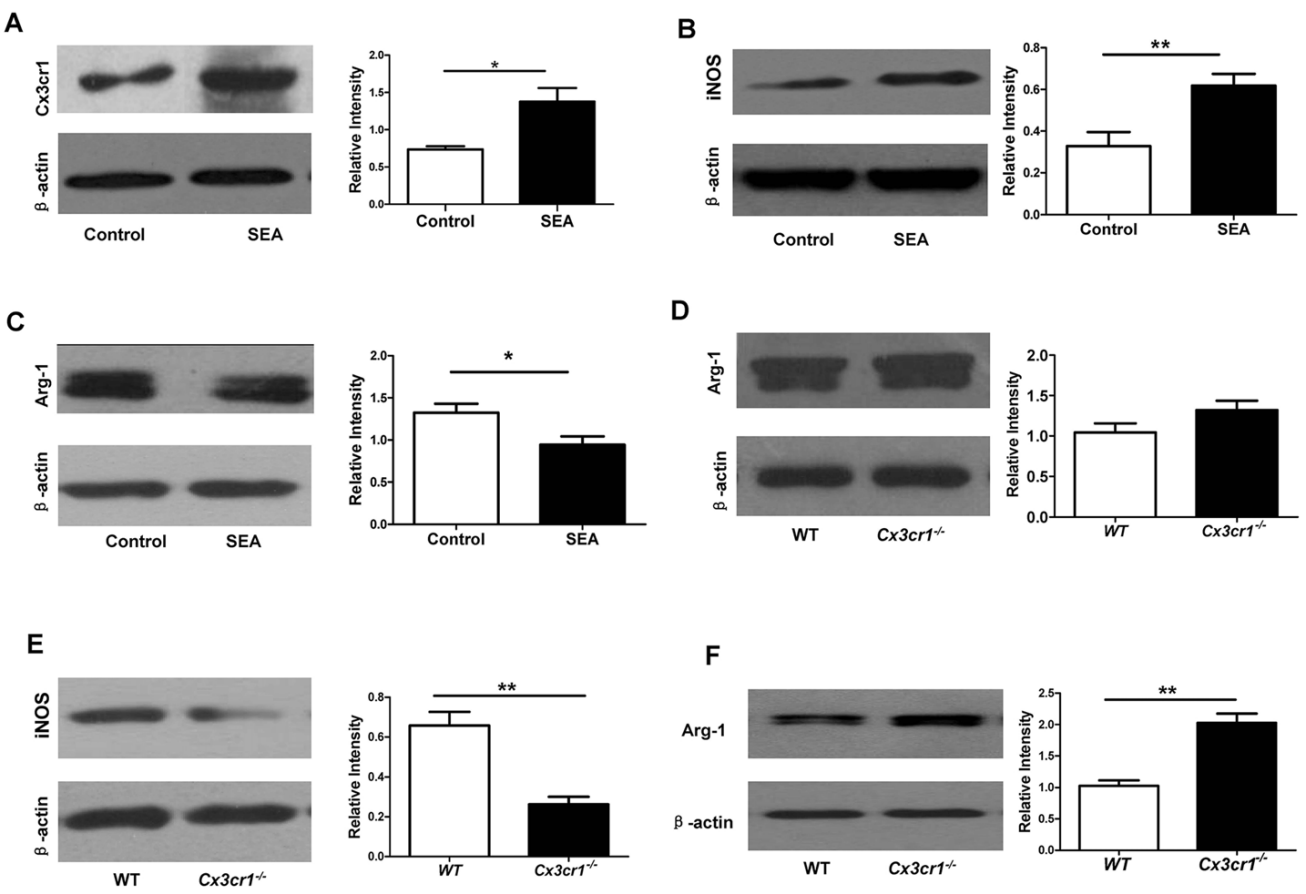
***Cx3cr1^−/−^* macrophages manifest enhanced Arg-1 and repressed iNOS expression after SEA stimulation.** (A) SEA stimulation induced expression of significantly higher levels of Cx3cr1 in macrophages. (B) SEA induced a 1-fold increase of iNOS expression in macrophages. (C) Addition of SEA significantly attenuated Arg-1 expression in macrophages. (D) *Cx3cr1^−/−^* macrophages manifested a slightly higher, but not a statistically significant, Arg-1 expression as compared with WT macrophages before SEA stimulation. (E) Loss of *Cx3cr1* resulted in a 1.5-fold reduction of SEA-induced iNOS expression in macrophages. (F) Macrophages deficient in *Cx3cr1* manifested a 1-fold higher Arg-1 after SEA stimulation. *P<0.05; **P<0.01.

Next, we compared the expression of Arg-1 and iNOS between WT and *Cx3cr1^−/−^* mice after *S. japonicum* infection. Remarkably, significantly higher levels of Arg-1 were noted in *Cx3cr1^−/−^* mice than in WT mice (Fig. 4C). By contrast, *Cx3cr1^−/−^* mice manifested a 4.8-fold reduction in iNOS expression as compared to WT mice (Fig. 4D). Of note, no significant difference in terms of Arg-1 (Fig. 4E) and iNOS expression (Fig. 4F) between WT and *Cx3cr1^−/−^* mice before *S. japonicum* infection was detected. Taken together, these data suggest that *Cx3cr1* deficiency promotes a characteristic switch of host immunity against *S. japonicum* eggs from a typical Th1 response to a Th2-based reaction during the stage of acute schistosomiasis.

### Lack of *Cx3cr1* preferentially induces macrophages toward M2 polarization

To confirm the above data obtained in animal studies, peritoneal macrophages were isolated from WT and *Cx3cr1^−/−^* mice, and then subjected to stimulation with SEA. SEA induced expression of high levels of Cx3cr1 in WT macrophages (Fig. 5A). In particular, SEA displayed a high potency to induce iNOS expression (Fig. 5B), whereas the expression of Arg-1 was repressed by SEA in WT macrophages (Fig. 5C). We then compared the expression of iNOS and Arg-1 between WT and *Cx3cr1^−/−^* macrophages. No perceptible difference in iNOS expression between WT and *Cx3cr1^−/−^* macrophages before SEA stimulation was noted (data not shown), whereas *Cx3cr1^−/−^* macrophages manifested a slightly higher Arg-1 expression as compared to WT macrophages (Fig. 5D). However, WT macrophages displayed a 1.5-fold higher iNOS expression than that of *Cx3cr1^−/−^* macrophages 96 h after SEA stimulation (Fig. 5E), and, by contrast, a 1-fold higher Arg-1 expression was detected in *Cx3cr1^−/−^* macrophages as compared with that of WT macrophages (Fig. 5F). Collectively, these data support that *Cx3cr1^−/−^* macrophages preferentially polarize to a M2 phenotype upon SEA induction.

**Fig. 6.**
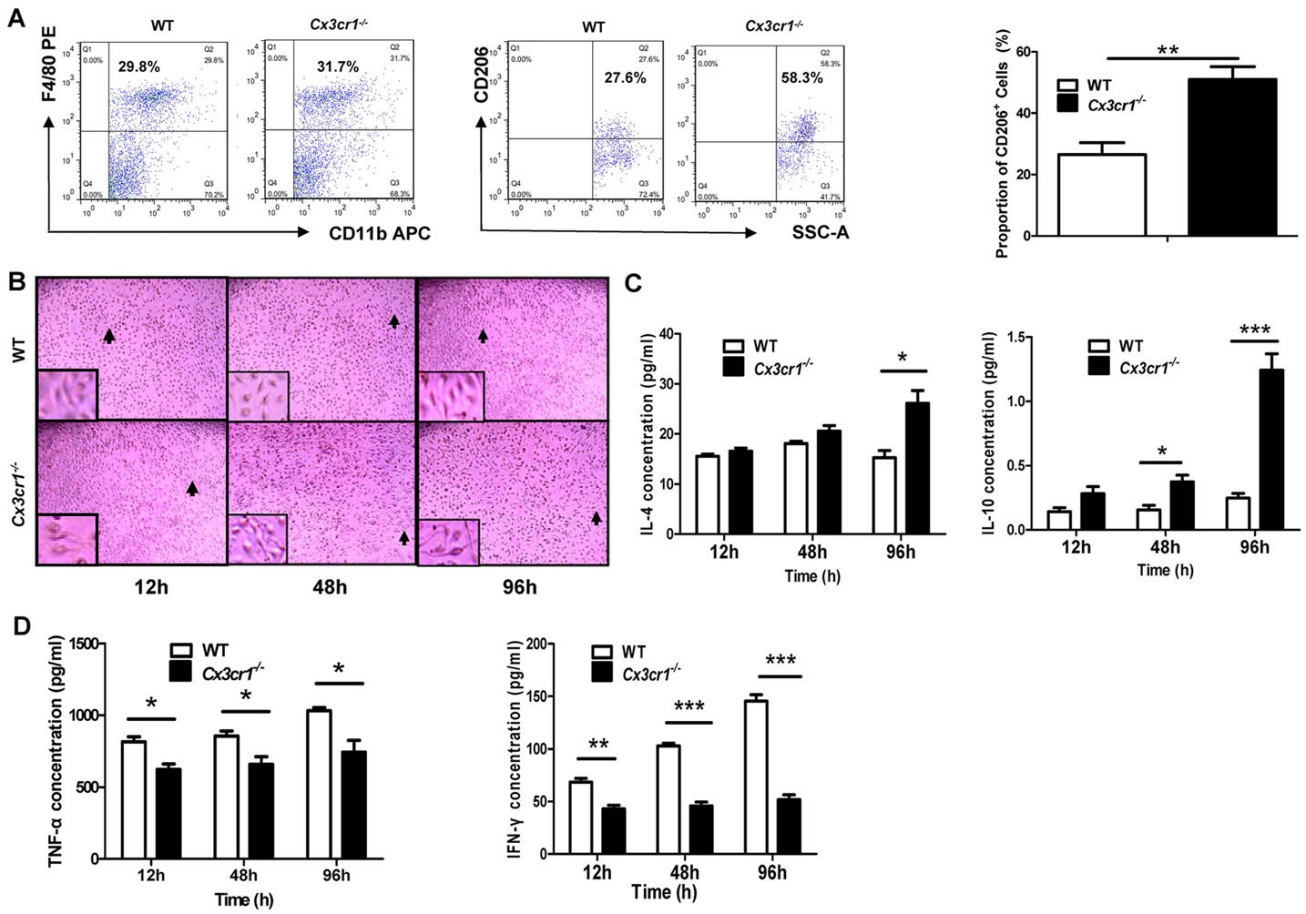
**Phenotypic analysis of *Cx3cr1^−/−^* macrophages after SEA stimulation.** (A) Flow cytometry analysis of CD206 expression in F4/80^+^ CD11b^+^ macrophages after SEA stimulation. *Cx3cr1^−/−^* macrophages manifested a significantly higher proportion of CD206^+^ cells as compared to WT macrophages. (B) Comparison of temporal morphological characteristics between WT and *Cx3cr1^−/−^* macrophages after SEA stimulation. The locations of the enlarged insets are indicated by arrows. (C) ELISA analysis of IL-4 and IL-10 secretion into culture supernatant after SEA stimulation. (D) The production of TNF-α and IFN-γ from macrophages after SEA stimulation. Three replications were conducted for all studies. *P<0.05; **P<0.01; ***P<0.001.

We next conducted flow cytometry for analysis of CD206 expression, another phenotypic marker for M2 macrophages (Martinez, 2011). For this purpose, SEA-stimulated macrophages were first gated for CD11b and F4/80, and CD11b^+^ F4/80^+^ cells were then subjected to analysis of CD206 expression. Indeed, there was an ∼1.0-fold higher number of *Cx3cr1^−/−^* macrophages that were CD206-positive as compared with the number of WT macrophages at 96 h after SEA stimulation (Fig. 6A). Moreover, SEA induced a distinctive morphological change in the macrophages (Lee et al., 2013). The most striking morphological differences were noted 96 h after SEA stimulation, in which WT macrophages displayed M1 morphological features, characterized by the hemispherical shape along with larger surface area, full volume and high tension, whereas *Cx3cr1^−/−^* macrophages demonstrated M2 characteristics of stellate shape featured by long tentacles and a small surface area as well as low tension (Fig. 6B). Of note, no perceptible morphological difference was noted between WT and *Cx3cr1^−/−^* macrophages before SEA stimulation (data not shown). These results prompted us to examine the differences in their cytokine secretion. To do this, we assayed TNF-α, IFN-γ, IL-4 and IL-10 production in the culture supernatants at various time points. In agreement with the above data, *Cx3cr1^−/−^* macrophages secreted significantly higher levels of IL-4 and IL-10 than WT macrophages (Fig. 6C), whereas WT macrophages secreted significantly higher levels of TNF-α and IFN-γ as compared to *Cx3cr1^−/−^* macrophages (Fig. 6D).

**Fig. 7.**
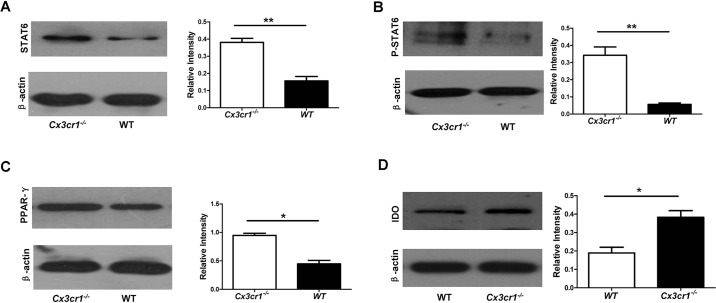
**Loss of *Cx3cr1* enhances STAT6** **and** **PPAR-γ signaling.** (A) Western blot analysis of total STAT6 in macrophage lysates after SEA stimulation. (B) Western blot results for phosphorylated STAT6 (p-STAT6). (C) Western blot results for PPAR-γ. (D) Western blot analysis of IDO in macrophages after SEA stimulation. *P<0.05; **P<0.01.

### *Cx3cr1* deficiency promotes STAT6–PPAR-γ signaling and IDO expression

We next sought to address how loss of *Cx3cr1* skews macrophages towards M2 polarization upon stimulation with SEA. Given that STAT6 has been demonstrated to act as an IL-4 signal mediator (Sajic et al., 2013), we thus first examined the impact of *Cx3cr1* deficiency on the STAT6-PPAR-γ axis, an essential signaling pathway relevant to the induction of M2 macrophages (Sica and Mantovani, 2012). As expected, SEA induced more than a 1.5-fold increase in total STAT6 in *Cx3cr1^−/−^* macrophages at 96 h after stimulation with SEA as compared to in control macrophages (Fig. 7A). In line with this observation, *Cx3cr1^−/−^* macrophages manifested significantly higher levels of phosphorylated (activated) STAT6 (p-STAT6) than control macrophages (Fig. 7B). Analysis of the downstream factor PPAR-γ also revealed that SEA-stimulated *Cx3cr1^−/−^* macrophages express much higher levels of PPAR-γ (Fig. 7C). To further demonstrate that the enhanced STAT6 and PPAR-γ signaling promotes *Cx3cr1^−/−^* macrophages towards M2 differentiation in the setting of acute schistosomiasis, we examined indoleamine 2,3-dioxygenase (IDO) expression, an immunosuppressive marker relevant to the functionality of M2 macrophages. Indeed, SEA induced a 1.0-fold higher IDO expression in *Cx3cr1^−/−^* macrophages than in control macrophages (Fig. 7D). Taken together, these data suggest that loss of *Cx3cr1* enhances IL-4 and IL-10 secretion, which then promotes STAT6 and PPAR-γ signaling to promote macrophages towards M2 polarization, as manifested by the high levels of IDO expression.

## DISCUSSION

During acute schistosomiasis worm ova released from adult *S. japonicum* elicit potent pro-inflammatory responses along with characteristic granuloma formation, which then causes substantial injuries to organs such as the liver and intestine where the eggs are trapped (Hirata et al., 2001). As a result, anti-inflammatory responses to limit excessive liver injury or intestinal hemorrhage are necessary to prevent host lethality (Herbert et al., 2008). In general, acute schistosomiasis is considered a Th1 disease (de Jesus et al., 2002), and a defect in developing a Th2 response during acute schistosomiasis is associated with high lethality in mice (Rani et al., 2012). Given that alternatively activated M2 macrophages possess a high capacity for secretion of Th2 cytokines, their role in limiting pro-inflammatory responses and granuloma formation during acute schistosomiasis has been highly appreciated.

Previous studies have suggested that the trafficking of monocytes and macrophages from peripheral blood into the sites of inflammation is a dynamic, multi-step process, and that Cx3cr1 plays a crucial role in the regulation of macrophage trafficking (Shi and Pamer, 2011). Indeed, Cx3cr1 has been found to promote atherosclerosis lesion by regulating macrophage accumulation (Lesnik et al., 2003; Poupel et al., 2013), and blockade of Cx3cr1 attenuates renal pro-inflammatory responses and fibrosis after ischemia-reperfusion insult (Furuichi et al., 2006). Based on these observations, we assessed the role of Cx3cr1 in hepatic granuloma formation in the setting of acute schistosomiasis by employing *Cx3cr1^−/−^* mice. Remarkably, loss of *Cx3cr1* significantly protected mice from liver injury and functional impairment mediated by *S. japonicum* infection, as manifested by reduced body weight loss, attenuated hepatomegaly and suppressed granuloma formation along with preserved liver function. Studies in WT control mice further revealed that *S. japonicum* infection induced high levels of hepatic Cx3cr1 expression, and the induced Cx3cr1 was predominantly expressed by the infiltrating macrophages. Taken together, our data support the hypothesis that that inhibition of Cx3cr1 signaling could be an effective approach to limit pro-inflammatory responses and granuloma formation during acute schistosomiasis.

For the first time, we demonstrated that loss of *Cx3cr1* causes macrophages to preferentially polarize to an M2 phenotype during *S. japonicum* infection. As discussed earlier, acute schistosomiasis is characterized by a Th1 response, and indeed, previous studies in WT mice have demonstrated a significant upregulation of iNOS, a particular marker for Th1-deviated responses during schistosome infection (Brunet et al., 1997). Interestingly, *Cx3cr1^−/−^* mice manifested an opposite phenotype, in which significantly higher levels of Arg-1, a typical marker for Th2 responses, were detected, whereas iNOS expression was significantly suppressed as compared to WT control mice. These data provide suggestive evidence supporting the idea that *Cx3cr1* deficiency skews macrophages preferentially towards M2 polarization during acute schistosomiasis. To confirm this assumption, we conducted studies in peritoneal macrophages that were stimulated with extracted SEA. Indeed, SEA induced a potent Th1 response in WT macrophages as manifested by the upregulation of iNOS expression along with enhanced TNF-α and IFN-γ secretion. However, a typical Th2 response was noted in *Cx3cr1^−/−^* macrophages, as characterized by the high levels of expression of Arg-1 along with increased IL-4 and IL-10 secretion after SEA exposure. Notably, flow cytometry analysis of CD206 expression, a marker associated with M2 polarization, revealed that the majority of macrophages were positive for CD206 after SEA stimulation. Taken together, our data demonstrate that *Cx3cr1* deficiency leads to a typical switch of the host immune defense from a conventional Th1 to a typical Th2 response during acute schistosomiasis.

It has been well demonstrated that control of macrophage polarization is largely attributed to the function of a small group of factors including STATs and PPARs (Sajic et al., 2013), and that, particularly, phosphorylation of STAT6 is a common signaling factor relevant to M2 polarization of macrophages (Mishra et al., 2011). We thus next examined STAT6 and PPAR-γ signaling to address the mechanisms by which *Cx3cr1* deficiency skews macrophages towards M2 polarization during acute schistosomiasis. In accordance with our expectation, SEA induced a significant increase in STAT6 expression in *Cx3cr1^−/−^* macrophages along with much higher levels of phosphorylated STAT6 (p-STAT6) as compared to in WT control macrophages. Similarly, significantly higher levels of PPAR-γ were detected in *Cx3cr1^−/−^* macrophages than WT control macrophages. We further noted that SEA stimulation resulted in a 1.0-fold higher IDO expression in *Cx3cr1^−/−^* macrophages than in control macrophages. Previous studies have demonstrated that IDO expression in macrophages serves as an important mechanism to limit the pro-inflammatory response in the setting of tissue and organ injury or infection (Kim et al., 2009), and defective IDO production from macrophages results in greater inflammatory infiltration along with excessive collateral damage to parasitized organs (Rani et al., 2012). Therefore, these data support the notion that loss of *Cx3cr1* promotes STAT6 and PPAR-γ signaling along with enhanced IDO expression, which then promotes M2 polarization of macrophages upon acute schistosome infection.

It should be noted that we only investigated the impact of *Cx3cr1* deficiency on the polarization of M2 macrophages in the setting of acute schistosomiasis, therefore, we cannot completely exclude the possibility that *Cx3cr1* deficiency also affects additional pathways. Furthermore, we only examined pro-inflammatory responses and hepatic granuloma formation during acute schistosomiasis; the impact of *Cx3cr1* deficiency on hepatic fibrosis in the setting of chronic schistosomiasis, however, is yet to be elucidated, which should be a major focus for future studies. Nevertheless, our present data provide evidence supporting the hypothesis that CX3CR1 could be a viable therapeutic target during schistosomiasis. In fact, disruption of CX3CR1 signaling has been utilized in proof-of-concept preclinical studies (Ishida et al., 2008). The first CX3CR1 antagonist with anti-inflammatory activity both in mice and humans was recently described (Dorgham et al., 2009). Selective CX3CR1 antagonists could be also beneficial for limiting chronic inflammatory process during *S. japonicum* infection.

In summary, we demonstrated that blockade of Cx3cr1 signaling provides protection for mice against pro-inflammatory responses and hepatic granuloma formation in the setting of acute schistosomiasis. Our mechanistic studies revealed that loss of *Cx3cr1* renders macrophages preferentially towards M2 polarization, which involves STAT6 and PPAR-γ signaling along with enhancement of IDO expression. Taken together, our data provide evidence suggesting that CX3CR1 could be a viable therapeutic target in the clinical setting of patients with acute schistosomiasis.

## MATERIALS AND METHODS

### Animals

C57BL/6 mice and B6 *Cx3cr1^−/−^* mice were obtained from the Jackson's Laboratory (Bar Harbor, ME, USA). The mice were housed in the SPF animal facility of Tongji Medical College, Huazhong University of Science and Technology in microisolator cages supplied with sterile food and water with a 12-h light and 12-h dark cycle. All animal procedures were performed in accordance with the National Institute of Health guidelines and were approved by the Animal Care and Use Committee at Tongji Hospital of Huazhong University of Science and Technology.

### Infection of mice with *S. japonicum*

Each mouse was infected with 30 cercariae of *S. japonicum* percutaneously through a shaved abdomen using the coverslip method as described previously (Maeda, 1982). Cercariae of *S. japonicum* originating from *Oncomelania hupensis* snails were purchased from the Jiangsu Institute of Parasite Disease (Wuxi, China). The mice were sacrificed 8 weeks after infection, and tissues and organs were collected for experimental purposes. Fifteen mice were included for each study group.

### Preparation of soluble egg antigen

Parasitic eggs were isolated from the liver of B6 mice 8 weeks after infection with cercariae by enzymatic digestion using 0.01% pronase and 0.05% collagenase (Sigma Chemical Co., St Louis, MO). The eggs were suspended in 4°C PBS and then homogenized on ice until more than 95% of the eggs were disrupted. The supernatants were collected and sterilized by passing through a 0.2-µm filter after ultracentrifugation.

### Histological analysis and immunostaining

The collected tissues and organs were first fixed in 4% paraformaldehyde overnight and then embedded in paraffin. Tissue sections (5 μm) were prepared using a Leica HM-325 rotary microtome, and then subjected to H&E staining as previously described (Zhong et al., 2014). For immunostaining, the sections were first deparaffinized in xylene and rehydrated in graded alcohol. Nonspecific proteins were blocked with 10% goat serum or rabbit serum for 30 min. The sections were then probed with a rat-derived anti-F4/80 antibody (Serotec, Raleigh, NC, USA; 1:100) and a rabbit-derived anti-CX3CR1 antibody (Abcam, Cambridge, MA; 1:100) at 4°C overnight, followed by incubation with an Alexa-Fluor-546-labeled anti-rat-IgG and an Alexa-Fluor-594-labeled anti-rabbit-IgG secondary antibody (Invitrogen, Carlsbad, CA; 1:500) at room temperature for 30 min as previously reported (Rao et al., 2011). Sections stained with normal IgG were used as a negative control. Immunofluorescence images were acquired using a scanning sequential mode to avoid bleed-through effects with a fluorescence confocal microscope (Carl Zeiss LSM 710, Germany), and images were processed using the ZEN 2009 software (Carl Zeiss, Germany).

### Assessment of hepatic granuloma formation

Hepatic granuloma area was assessed in H&E-stained sections originating from each group of mice, and 30 granulomas were included for each group of mice. The granuloma size (μm^2^) was defined by the area containing a single schistosome egg, and liver sections from 8 mice in each group were randomly chosen for statistical analysis of granuloma areas. The sections were evaluated under an Olympus AX-80 microscope, and images were taken using an Olympus DP 71 camera. Granuloma area was then calculated using the IMAGE PRO PLUS software package v. 6.0 by tracing the border of granulomas. An experienced pathologist evaluated all sections in a blinded fashion, and the severity of inflammatory infiltration was also assessed.

### Peritoneal macrophage isolation, culture and treatment

Peritoneal macrophages from both wild-type (WT) B6 and *Cx3cr1^−/−^* mice were isolated as previously reported (Zhong et al., 2010). Briefly, the mice were intraperitoneally injected with 5 ml sterilized cold RPMI 1640. Peritoneal macrophages were harvested by washing peritoneal lavage twice with 5 ml cold RPMI 1640. After lysis of red blood cells, the cells were incubated for 3 h at 37°C in 35-mm×15-mm tissue culture dishes. Non-adherent cells were removed by exhaustive washing with 1× PBS. Viability of macrophages was assessed by the Trypan Blue exclusion method. The adherent macrophages were next plated at a density of 1×10^6^ cells/ml followed by stimulation with 1 µg of SEA. The cells and culture supernatants were collected for experimental purpose 96 h after addition of SEA.

### ELISA analysis of cytokine production

The levels of TNF-α, IFN-γ, IL-4 and IL-10 in the serum samples and culture supernatants were measured with enzyme-linked immunosorbent assay (ELISA) kits purchased from BD Biosciences (San Jose, CA) using the established techniques within the laboratory (Han et al., 2008).

### Western blot analysis

Liver tissues and cultured macrophages were homogenized in RIPA lysis buffer (Beyotime, Shanghai, China) using a BBX24 Bullet Blender homogenizer (Next Advance Inc., Averill Park, NY) according to the manufacturer's instruction. The lysates were separated by 10% SDS-PAGE and then transferred onto polyvinylidene difluoride (PVDF) membranes. Western blot analysis was carried out as reported previously by probing the blots with the indicated primary antibody followed by a horseradish peroxidase (HRP)-conjugated secondary antibody (Yang et al., 2013). The reactive bands were visualized using an ECL Plus Western Blotting kit (PIERCE, Rockford, IL) according to the manufacturer's instructions. Primary antibodies against CX3CR1 (1:1000), indoleamine 2, 3-dioxygenase (IDO, 1:1000), STAT6 (1:500), PPAR-γ (1:500), and β-actin (1:500) were obtained from Abcam (Cambridge, MA), whereas antibodies against iNOS (1:250) and Arg-1 (1:4000) were purchased from BD Pharmingen (Carlsbad, CA). The intensity of each reactive band was analyzed using the densitometry plugin ImageJ software (http://rsb.info.nih.gov/ij/).

### Flow cytometry analysis

Surface marker expression on macrophages 96 h after SEA stimulation was determined by flow cytometry as previously reported (Han et al., 2008). Briefly, macrophages were first washed twice with FACS medium (2% heat-inactivated FCS in PBS), followed by incubation with phycoerythrin-labeled F4/80 (eBioscience, Danvers, MA), APC-labeled CD11b and FITC-labeled CD206 (BD Pharmingen, Carlsbad, CA) at 4°C for 30 min. After washes, the cells were subjected to flow cytometry analysis, and all data were analyzed using the FACSCalibur and FlowJo version X software.

### Statistic analysis

All data are shown as mean±s.e.m. All *in vitro* experiments were conducted with three independent replications. Graphpad Prism 5 was used for statistical analysis using Student's *t*-test or one-way or two-way ANOVA and Bonferroni's post hoc test where appropriate. In all cases, *P*<0.05 was considered statistically significant.
